# In Its Southern Edge of Distribution, the Tawny Owl (*Strix aluco*) Is More Sensitive to Extreme Temperatures Than to Rural Development

**DOI:** 10.3390/ani12050641

**Published:** 2022-03-03

**Authors:** Orr Comay, Efrayim Ezov, Yoram Yom-Tov, Tamar Dayan

**Affiliations:** 1 School of Zoology and the Steinhardt Museum of Natural History, Tel Aviv University, Tel Aviv 6997801, Israel; yomtov@tauex.tau.ac.il (Y.Y.-T.); dayant@tauex.tau.ac.il (T.D.); 2 Mount Meron Field School, The Society for the Protection of Nature in Israel, Upper Galilee Regional Council, 1387000, Israel; ezov1234@walla.co.il

**Keywords:** climate impacts, diet, edge of distribution, Israel, nesting, ontogeny, species distribution

## Abstract

**Simple Summary:**

Tawny Owls occur in a wide range, with Israel being the southernmost country where they occur. This country’s climate is warming rapidly and it also undergoes an extensive urban and agricultural development. However, it is unknown to what extent these processes would endanger the Tawny Owls living there. To gain insight about this question, we surveyed Tawny Owls in the field, tracked the number of hatchlings in their nests, and also analyzed how their diet varied between different environments. We trained models of Tawny Owl distribution, number of hatchlings and diet using climate and land use variables to figure out to what extent climate change and development impact this species’ Israeli population. We found that Tawny Owls prefer relatively cool, rainy and wooded areas within Israel, and occur more often in villages compared to open fields. Tawny Owls raised more hatchlings in pine forests, especially when spring temperatures were moderate and following rainy winters. Tawny Owls preyed predominantly on Günther’s Voles everywhere, but took more birds in rural environments compared to forests. Our results suggest that climate change, which would increase spring temperatures and decrease rainfall, is a larger threat to Tawny Owls in Israel than rural development.

**Abstract:**

Populations at the warm edge of distribution are more genetically diverse, and at the same time are more susceptible to climate change. Between 1987–1996, we studied Tawny Owls in Israel, the species’ global southern edge of distribution and a country undergoing a rapid land cover transformation for over a century. To assess the potential impacts of land cover transformation, we modelled the species’ most suitable habitat and climate and analyzed how climate and habitat affected the nesting success and prey selection. Moreover, we monitored Tawny Owl juveniles’ survival and ontogeny from eggs to dietary independent young, to find out whether the Israeli population is a sink. While the species distribution model correctly predicted the Tawny Owl’s densest areas of occurrence, it failed to predict its occurrence in adjacent regions. The model also predicted that areas included in the species’ historical range remained suitable habitats. The number of fledglings increased with precipitation and in rural settings but was adversely affected by extreme temperatures. While voles dominated the diet in all habitats, the Tawny Owl’s diet is considerably more variable than other Israeli owls. Our results suggest that the Tawny Owl can adapt to rural-agricultural environments, but is susceptible to climate change.

## 1. Introduction

Species’ edges of distributions at higher latitudes and altitudes shifted with the Quaternary’s climatic changes, with populations at the warmer edge being the source of expansion during interglacial periods and persisting during glacial periods [1]. Hence, populations at the warmer edge of species’ distribution are considerably older, more genetically diverse and distinct from those at the cooler edge, making them disproportionally important to species conservation. In Israel, current global warming (leading to warmer climates than previous interglacials) is predicted to shift distributions of montane species northward [2], thus threatening these important populations. It is therefore of interest to study the natural history of populations at the warmer edge of distribution and analyze to what extent their natural history differs from those at the core of distribution.

**Figure 1:**
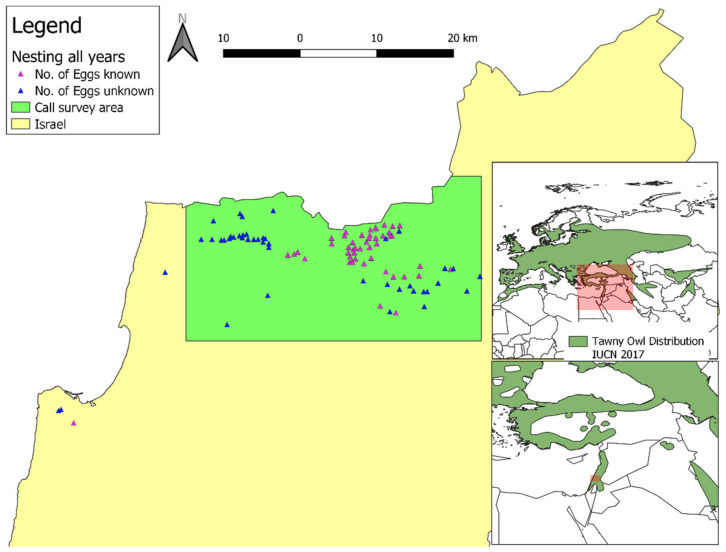
Map of the study area, with nesting sites and the area surveyed for male territorial calls. The global distribution of the Tawny Owl (*Strix aluco*; in dark green) is from [3]. IUCN stands for International Union for Conservation of Nature. Reprinted with permission from ref. [3]. Copyright 2016 IUCN.

Here we present a thorough study on the natural history of the Tawny Owl (*Strix aluco*) in Israel, at its southern edge of distribution in the Palearctic [3] (Figure 1). Tawny Owls are known to inhabit woodlands, but also human settlements, including urban parks [4]. In Israel, they nest in tree cavities, but sometimes also in artificial constructions such as barns, towers and nesting boxes (pers. obs.). In addition to the threat presented by climate change, Tawny Owl populations in Israel could also be stressed by the rapid land cover transformation [5]. Between 1881 and 2011 (a period which saw the rise of Zionism, the establishment of the State of Israel and consequent rapid development and fast demographic growth), the total built up area in Israel increased by more than 70-fold and the total area of orchards, vineyards increased by about 3-fold, while the total area of marsh lands, winter ponds, garrigues and vegetated dunes decreased by 93.4%, 70.4%, 59.2% and 32.0%, respectively. During the same time, the total area of conifers increased by more than 100-fold and that of Mediterranean maquis increased by 21.6%. Between the years 1950 and 2017, the mean annual temperature in Israel increased by 1.4 degrees Celsius [6], and it is expected to further increase by another 1.5–3.7 degrees Celsius by the end of the 21st century [7]. The 20th century saw the Tawny Owl’s extirpation from the Judean Mountains and southern Samaria [8], effectively contracting its range, that moved ~60 km northwards to the Sharon region.

Given the dual threat of extensive land cover transformation and rapid climate change, it is imperative to understand to what extent the Tawny Owl can adapt to these changes and survive. We analyze how distribution, reproduction, and diet are driven by a combination of land cover and climate, in order to estimate to what extent the observed and projected trends in the environment will impact Tawny Owl conservation. We hypothesize that hot weather extremes would have a negative impact on Tawny Owls in their warm edge of distribution, while the species would prove resilient to cold weather extremes (in local terms). We also expect to find more commensal prey species in rural areas, and more Tawny Owls in general in wooded habitats. In addition, we describe egg to adolescent survival rates in the Israeli population and compare them to those known from elsewhere in the species’ range, in order to assess whether this southern population can be a sink (e.g., if survival rates are extremely low) that is dependent on immigrants. Establishing a species’ regional population as a sink causes the species to obtain a worse conservation status for that region [9].

## 2. Materials and Methods

We studied the distribution, nesting success, and diet of Tawny Owls in Israel. Fieldwork was conducted during the years 1987–1998 (see below for details).

### 2.1. Study Area

We surveyed Tawny Owl calls (see below) in the Upper Galilee (Figure 1), defined here as the area from latitude 32.9° N in the south to the Israel-Lebanon border and latitude 33.13° N (the southern of the two; i.e., the study did not include Lebanese territory) in the north, longitude 35.16° E in the west to longitude 35.57° E in the East (some 817 km^2^). We have made efforts to ensure that this area was surveyed thoroughly in its entirety (see below). We used the calls recorded in this survey to train a species distribution model (SDM; see below). In addition to the formal survey, we recorded observations of Tawny Owls (mostly male territorial calls) made in Israel, including museum specimens (The Steinhardt Museum of Natural History and The National Natural History Collections), observations by birders, trackers, farmers, the Israel Wildlife Hospital’s staff and the general public. We used these observations to test the SDM and not to train it.

### 2.2. Field Survey

We thoroughly surveyed the Upper Galilee for Tawny Owls during the years 1987–1990. Field trips took place throughout the year and included all land uses and terrain types, including nature reserves, cultivated fields and plantations, rural and urban settlements, mountains, seasonal streams (wadis) and valleys. We located listening positions at about 1 km away from each other in a grid covering the entire study area, assuming one could hear a Tawny Owl call from about this distance in this mountainous terrain. We visited each listening position at least once during the late winter and early spring (when call frequency was deemed highest), but some locations were deemed suitable for the installation of nesting boxes and these were visited more often (see below). In early visits, we played a recording of Tawny Owl calls and recorded any response from Tawny Owls. Nevertheless, due to the unwieldiness of the playback equipment in field conditions, in later visits we sounded a human vocal imitation of Tawny Owl calls, with no observable differences in response rates. We recorded all observations in Tawny Owls, including detailed information of time of observation (start and finish), age (adult/fledgling), sex and activity (calling, flying, nesting etc.), as best as could be determined in the field. We did not record absences, resulting in a presence-background dataset (*sensu* [10]).

### 2.3. Species Distribution Model

SDMs require predictors (spatial environmental data) and observation data. During the field survey in 1987–1990, we were not aware of the importance of recording absence data for species distribution models, and thus we have presence-background data rather than presence-absence data. Given this nature of our survey data, we used the MaxEnt (Maximum Entropy) algorithm [11], which was designed to handle presence-background data, to create the SDM. A total of 126 observations were used to train and test the MaxEnt model (Table 1). The predictors we chose included both climate and land cover variables, because previous research on the use of SDMs to model bird ranges stressed that both are important for a reliable fit [12]. We downloaded mean annual temperature (tenths of degrees Celsius), altitude (meters above sea level) and mean annual precipitation (mm) raster data for 1950–2000 from worldclim.com [13]. Additionally, we used the vegetation index [14] as a predictor for the SDM; the vegetation index is an index of the Mediterranean botanic succession stage, ranging from 0 (built up), through 1 (cultivated fields and grasslands) and 2 (shrublands) to 3 (maquis or forest) for each land cell. Cell size was set to 0.00107° on 0.00107° (World Geodetic System 1984 coordinate system; approximately 119 m latitudinal distance on 100 m longitudinal distance in our study area). This relatively fine resolution allowed us to account for sharp climatic and land cover changes (e.g., between mountain peaks and valleys).

The observation data we used as input (training data) were only those we collected in the field survey (see above) and included only male territorial calls to control for detectability. Later observations and those made by others were only used to evaluate the resulting model, not to train it. That way, we controlled for observer skill and sampling effort (following [15]), allowing us to use the SDM to predict relative likelihood of Tawny Owl occurrence (i.e., where Tawny Owls are most likely to be found relative to other locations in the study area, regardless of their absolute abundance)–the most accurate result obtainable from presence-background data [10].

We divided the observations to four geographically exclusive subsets, based on latitudinal bands, and trained four models, each time excluding a single latitudinal band from the training set and using it as a test set instead (Table 1). This was done to avoid over-estimation of model performance caused by spatial autocorrelation, as occurs when test sets are chosen at random from the same area as the training set [16]. Each model predicted the relative likelihood of occurrence in a different latitudinal belt, where the observations of the Tawny Owls were withheld from the model. This resulted in four maps (one for each model and its respective test latitudinal belt) of predicted relative likelihood of occurrence, grading cells from 0 (least likely to find Tawny Owls, compared to other cells) to 1 (most likely to find Tawny Owls). For example, the map of the latitudinal belt 32.8°–32.98° N was created by a model that was trained on presence-background data from the latitudes of 32.98°–33.3° N (Table 1). We then merged the four predictions maps into a single map.

Afterwards, we trained another MaxEnt model on Tawny Owl and environmental data from the whole study area, and projected it to raster data of Mediterranean Israel and Golan Heights (hereafter ‘the combined model’). This resulted in predicting the relative likelihood of Tawny Owl occurrence in that area. Finally, we mapped the database of Tawny Owl observations collected outside the official field survey (see above) on top of the prediction map of the combined model in order to visually inspect the model’s performance. The area under the receiver-operator curve (AUC) is defined for our models as the probability that a random presence point will be ranked as more likely to host Tawny Owls than a random background point [17]. Notably, this metric is different from the absolute probability that Tawny Owls will be found in a given cell.

### 2.4. Nesting Survey

We installed nesting boxes for Tawny Owls in early 1988 (preceding the nesting season), focusing on areas where natural nesting sites (e.g., tree cavities and caverns) were lacking. In these regions, the alternative for Tawny Owls was to lay in buildings such as watch towers, in which nesting success was perceived as low. We tracked Tawny Owl nesting success in the Upper Galilee, including both nests in nesting boxes and elsewhere (trees, caves, ruins, structures etc.), during the years 1987–1996 (Figure 1). Overall, we tracked the number of eggs and hatchlings in 105 different nesting attempts, not including visits to nesting attempts where we do not know how many eggs were laid (e.g., when we visited for the first time that season only after the hatchlings hatched and could not approach the nest without disturbance) and visits to sites where nesting was attempted but no eggs were laid. We visited each nest at least once per nesting season (March to July, including the period when the parents feed their fledglings), and recorded the number of eggs and fledglings. When possible, we calculated the laying date based on the developmental stage of the chicks and assuming Tawny Owl chicks in Israel hatch after 28–29 days of incubation and fledge after 30–35 additional days [18]. Supplementary Material S1 is the full nesting database. Sites’ names and coordinates were omitted to prevent harassment of the Tawny Owls.

### 2.5. Ontogenetic Survey

In the years 1988–1989, we visited several nests multiple times (each time for a few hours) throughout the nesting season in order to track the ontogenetic development of the chicks, as well as to describe the survival curve of juvenile Tawny Owls, from eggs to independently hunting individuals. In addition to repeated visits, we also trapped three Tawny Owls (one adult male, one adult female and one young female) using lasso traps, in which live mice (*Mus musculus*) in a cage are used as bait. On top of the cage, lasso rings of wires are installed. Once an owl lands on the cage, there is a chance its leg would be trapped in a wire ring. We visited the traps every hour, and installed radio-tracking devices (model: SR-1 manufactured by Biotrack Ltd., Wareham, UK) on trapped Tawny Owls to follow them during chick-rearing season. Overall, 13 nesting attempts were tracked for the ontogenetic survey.

We defined three major ontogenetic milestones, and recorded at what age (in days) the chicks reached each milestone. When some of the chicks in the nest reached a given milestone and some did not, we assumed that the older ones reached it before their younger siblings. We defined the “fledging” milestone as the presence of a chick outside the nest cavity (from which point onward it is referred to as “fledgling”). We defined the “nest leaving” milestone as the first time a fledgling was recorded more than 150 m away from the nest, as an indication of greater mobility skills. We defined the “adolescence” milestone as the first time a fledgling ceased been fed by its parents. In practice, we indicated this milestone when a fledgling’s food calls were not heard anymore. In a single instance we also recorded this milestone when a fledgling kept calling for food, yet its parent chased it away rather than fed it. However, we assumed that a chick must reach a given milestone before reaching the next; hence, if a fledgling was not observed leaving the nest and was not heard calling its parents for food, we assumed it was dead (although no carcass was found) and not an adolescent.

As the nests were not visited every day, the exact age in which ontogenetic milestones were reached could not be recorded. For example, if at 5 May a chick was not observed outside its nest, and the next time it was observed at 9 May it was already outside its nest, it could have fledged at any day between 6 May and 9 May (including). Therefore, we recorded the minimal and maximal age of each milestone, when the minimal was defined as the chick’s age the day after the last time it was known not to have reach the milestone and the maximal as its age at the day it was observed to have reach the milestone. In the previous example, the minimal fledging age would be its age at 6 May and the maximal age would be its age at 9 May. Supplementary Material S2 is the ontogenetic dates database.

### 2.6. Diet Analysis

On several occasions, we collected and analyzed 209 Tawny Owl pellets from 9 different sites. We identified prey species based on morphology [19] and calculated the minimum number of individuals based on skeletal elements, teeth and feathers. We compared the prey composition in three dominant habitat types: rural (including both agriculture and villages), maquis and a mix of maquis and planted pines. For statistical analysis, we pooled prey count data from all sites having identical habitats. Supplementary Material S3 is the diet database.

### 2.7. Statistical Analysis

We created the MaxEnt SDMs using the software “MaxEnt” version 3.4.1 [11]. We used PAST [20] version 3.14 for the dietary analysis and the R programming language ([21]; version 3.5.0) for all other statistical analyses.

We used brood size as a proxy for nesting success, while clutch size was used as an offset to the models. We omitted from the analysis potential nesting sites where no eggs were found during the survey, rather than treating them as nests with a clutch and brood sizes of zero, even when other field signs indicated that nesting was attempted (e.g., feathers). Similarly, we omitted from the analysis nests where clutch size was unknown (e.g., as they were surveyed late in the season and only chicks were observed). We tested the effects of the land use (binary presence/absence of pines, oaks, and rural settlement/agriculture), and meteorological data for each year on Tawny Owl nesting success (brood size). The meteorological factors were the number of hot days (days in which the maximum temperature was higher than 30 °C in Safed, a city in the Upper Galilee; altitude ~850–900 m above sea level), maximum and minimal temperatures measured in Safed during March to May at that year and annual precipitation of the hydrological year (from September to August of the following year) at each nest and year. We calculated the latter from the Israeli Meteorological Service archive (Israel Meteorological Service 2017) using Kriging (a method predicting values in a spatial grid based on point data, when values are assumed to be more similar to nearer observations; [22]) from nearby rain gauges with the “automap” package version 1.0–14 [23]. We used the package “MASS” [24] to create generalized linear models of brood size as a function of environmental predictors. We included clutch size and the year as offsets (meaning that their impact was accounted for yet not tested for significance), because nesting surveys in later years focused on relatively successful nests (Table 2) and some failing nesting boxes were removed. We created models for two datasets: a) including all nesting sites where both clutch and brood sizes were known (N = 105; Table 3), and (b) a subset of the (a) in which the laying date of the first egg could be estimated (N = 94; see above). This allowed us to test the importance of laying date. We modelled each dataset twice with all predictors, once assuming a negative binomial distribution of brood size and once assuming a Poisson distribution. We used Akaike Information Criterion (AIC) to compare model fit within subsets. Once the full model was fitted, we dropped one predictor at a time and compared AIC with the full model to estimate the best fitting model (lowest AIC).

### 2.8. Relative Fitness in Warm Edge Populations

In order to test whether fitness (egg and offspring survival, nesting success and associated diet in habitats where survival or nesting success was relatively high or low) lower in Israel compared to populations closer to the species’ center of distribution, we compared our results with those obtained in similar studies from Europe. We used the mean values of the results summarized by [25] and by [26], as well as those reported by [27] and by [4]. In order to compare diet, we used the weights of living vertebrate prey from the literature [18,19,28] to calculate percentage of prey mass instead of by number of prey individuals.

## 3. Results

### 3.1. Species Distribution Model

**Figure 2:**
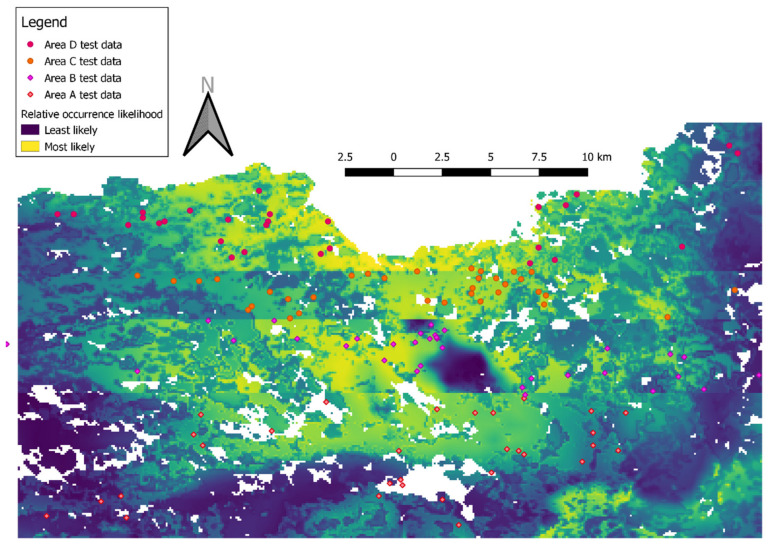
A merged map of four MaxEnt models of Tawny Owl (*Strix aluco*) relative likelihood of occurrence (cloglog output). The map is composed of four different MaxEnt cloglog outputs, each set to a latitudinal belt (Table 1) that was excluded from its training set. Diamonds and circles indicate observations of male territorial calls used as test data.

The combined map of relative likelihood of Tawny Owl occurrence (Figure 2) depicts the four models’ predictions in their respective test areas, as well as the actual observations in these areas. Yellow hues reflect higher predicted relative likelihood of Tawny Owl occurrence. White areas were excluded from the model, either because they were outside the study area (i.e., south of latitude 32.9° or in Lebanon) or because no vegetation index values were available for them. AUC values for the four models’ (A–D) training sets were 0.715, 0.749, 0.705 and 0.726, respectively.

**Figure 3:**
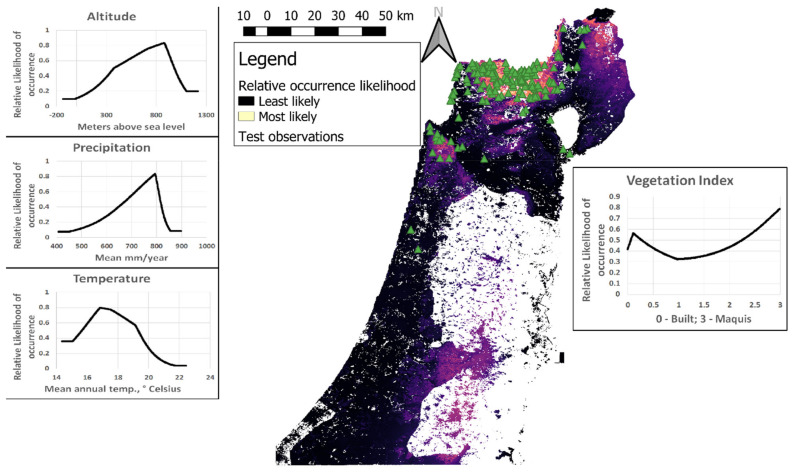
The cloglog output of the combined MaxEnt model, trained on all observations and predictors of the entire male territorial call survey area. Area shown includes Israel and the Golan Heights. Green triangles are observations in Tawny Owls (*Strix aluco*) taken outside the survey and were not used to train the MaxEnt model. Line plots depict the response curves of single predictors when used to predict Tawny Owl distribution in isolation (as sole input variables).

Overall, most observations were made in areas of higher relative likelihood of Tawny Owl occurrence and vice versa (Figure 3). Predictor variables had similar influences in all models A-D: altitude 700–900 m above sea level; mean annual temperature 16.5–17.5 °C; mean annual precipitation 750–800 mm; and vegetation index with two peaks, a short one (~0.43–0.65 on the cloglog scale (i.e., relative likelihood of occurrence;) on built areas and a taller one in the Mediterranean maquis (~0.73–0.81 on the cloglog scale).

The vegetation index had considerably lower training gain contribution (i.e., it contributed the least to differentiating between presence and background points; see [17] for further discussion) than all other predictors (~29%–51% of total model gain in models A-D), suggesting its inability to predict Tawny Owl relative likelihood of occurrence by itself. Nevertheless, in all four models the vegetation index was also the variable whose omission decreased the gain the most, suggesting it contains the most information uncontained in the other predictors (altitude, mean annual precipitation and mean annual temperature). In other words, the vegetation index had the lowest correlation with the other predictors (compared with other correlations between predictors).

### 3.2. Nesting Success

We surveyed a total of 105 nesting attempts in the years 1987–1996, in both natural sites and artificial nesting boxes. We report descriptive statistics in Table 2 and Table 3. In nests where the laying date was known (or could be calculated based on the hatchlings’ age during monitoring; n = 115), the mean laying date was ~24 March (rounded to the 84th day of the year), with a standard deviation of 12.34 days. The earliest laying date was 1 March and the latest was 26 April. In an analysis of nests whose laying date was known or calculated, laying date did not have a significant effect on the number of fledglings (*p* = 0.512). Therefore, the results reported hereafter relate to the full dataset, including all nests monitored, and laying date was omitted as predictor.

**Figure 4:**
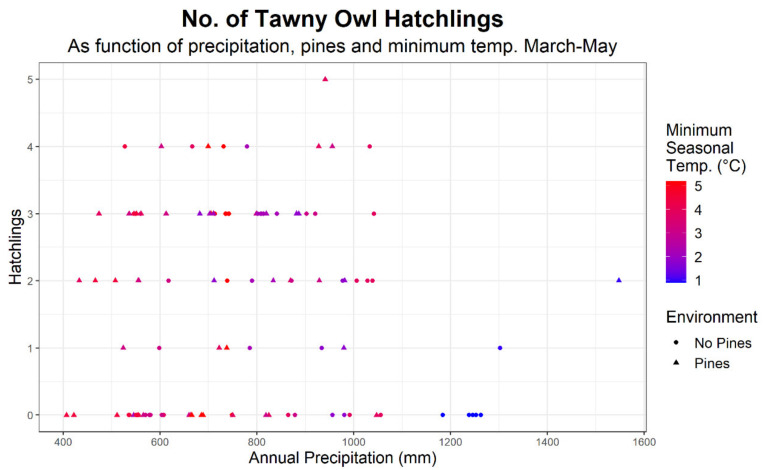
Number of Tawny Owl (*Strix aluco*) hatchlings as a function of Kriged precipitation at a given year-nest combination and the minimal temperature at Safed (Upper Galilee) during March-May of that year.

The following predictors had a significant positive effect on brood size: precipitation (log10-transformed, in mm), presence of pines and minimal temperature in March to May (i.e., brood size increased with the minimal temperature). The maximum temperature in March to May had a significant negative effect on the number of fledgling (i.e., hotter seasonal maximums are associated with lower nesting success). Raw and standardized predictor covariate values are given in Table 4 (only the standardized coefficients are directly comparable as they do not depend on the units (e.g., temperature in °C vs. log_10_(precipitation in mm)). Figure 4 depicts brood size as a function of precipitation and presence or absence of rural environment.

### 3.3. Juvenile Survival and Ontogeny

**Figure 5:**
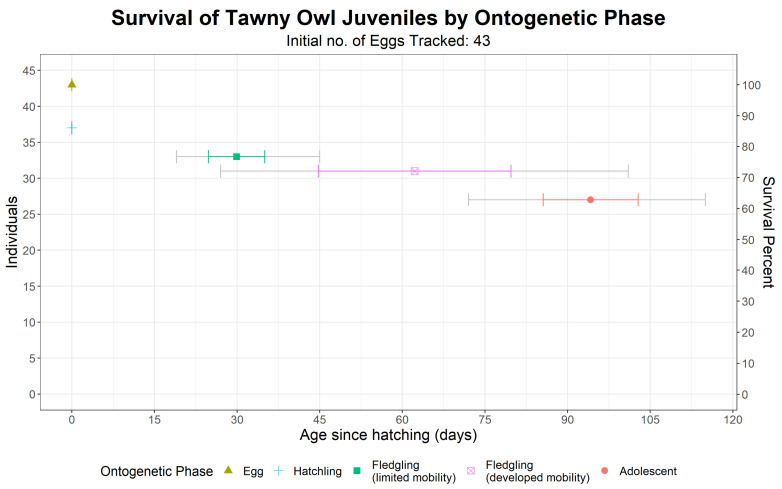
Number of surviving of Tawny Owls (*Strix aluco*) juveniles in each ontogenetic event and their mean age. Colored error bars are standard deviations, while grey error bars are the youngest and oldest ages. As not all individuals were surveyed daily for survival and ontogenetic phase, age data (means, standard deviations, youngest and oldest ages) include both minimal and maximal possible ages (see the Methods section).

We monitored the ontogeny and survival of a total of 43 eggs from 13 different nests (including two nesting sites that were used both in 1988 and in 1989) until adolescence (Figure 5). Of these, 37 eggs hatched, 33 hatchlings fledged, 31 fledglings left the nest vicinity and 27 reached adolescence. The overall survival rate was 62.8% (27 out of 43), with the highest mortality occurring at the egg phase: six out of the 16 individuals that did not survive until adolescence never hatched. Ontogenetic ages are reported in Figure 5.

### 3.4. Diet Analysis

**Figure 6:**
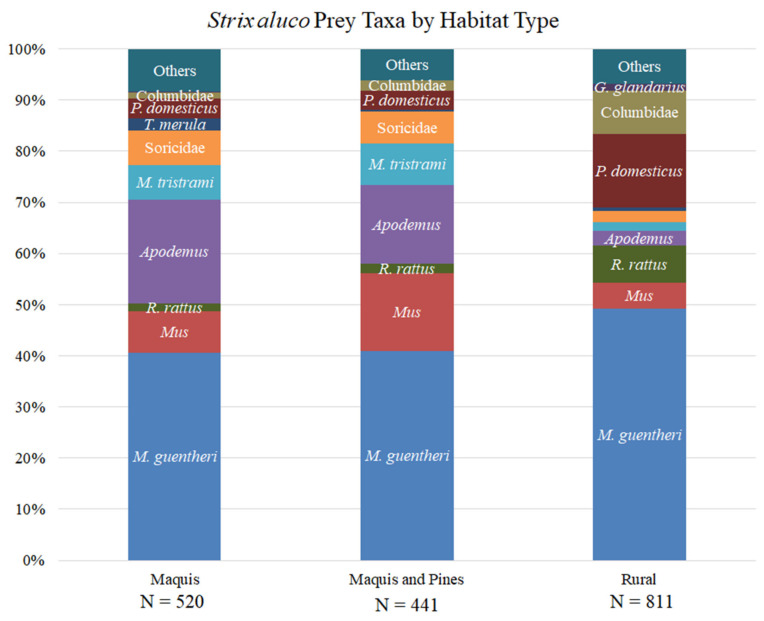
Tawny Owl (Strix aluco) diet per habitat type. Abbreviations: P. domesticus = Passer domesticus. T. merula = Turdus merula. M. tristrami= Meriones tristrami. R. rattus = Rattus rattus. M. guentheri = Microtus guentheri. G. glandarius = Garrulus glandarius.

Tawny Owls fed mainly on small mammals (79.6% of total prey items), especially in maquis and maquis-pine tree mixed stands (88–89% of prey items in these habitats, respectively). Günther’s Voles (*Microtus guentheri*) were the most common prey species (45% of total prey items), followed by field mice (*Apodemus* spp.; 11% of total prey items). We found highly significant differences between habitats in Tawny Owl prey composition (Monte Carlo with 9999 permutations *p*-value = 0.0001, χ^2^ *p*-value < 10^−4^). Prey taxa significantly more common in maquis included shrews (Soricidae), field mice and blackbirds (*Turdus merula*). In rural habitats, voles, rats (*Rattus rattus*), pigeons and doves (Columbidae) were significantly more common prey taxa compared to maquis and maquis and pines mixed stands. In maquis and pines mixed stands, jirds (*Meriones tristrami*), field mice and house and Macedonian mice (*Mus musculus* and *M*. *macedonicus*) were significantly more common prey taxa. Absolute prey taxa counts are given in Table 5, and their relative abundances are depicted in Figure 6. Table 5 also states in what habitat type each taxon was significantly more common, using the adjusted χ^2^ residuals threshold of ±3.

## 4. Discussion

We studied the distribution, fitness and diet of the Tawny Owl in its southern edge of distribution, an area threatened by rapid development and climate change.

While our field observations were collected in the late 20th century, there is no reason to doubt their relevance for the early 21st century: Our occupancy model, that was trained using decades old observations in Tawny Owls, was validated using recent observations; the nesting success model accounted for both rainy and drought years, and it was thanks to this variance that the effect of annual weather conditions was detected, especially that of extreme cold or hot spells during the spring. This insight would be useful in assessing the ecological impact of climate change on Tawny Owl conservation; the inter-annual variation in clutch and brood sizes was marginal within ten years of data and should provide a clear idea for these indices for the Israeli Tawny Owl population; while Tawny Owl diet was demonstrated to vary somewhat between years [29], the overall patterns (e.g., what are the dominant prey species and in what habitats they are more commonly taken) should stay consistent between years.

Some of the independent observations of Tawny Owls (including male territorial calls) appear in the areas least expected by the combined MaxEnt model (Figure 3). These include observations along the Galilee coast (northwestern Israel), the Hula Valley (northeastern valley), Jezreel Valley (northern Israel, east of Mount Carmel) and at the Sea of Galilee (northeastern Israel). Notably, these observations were taken in areas where the environmental predictor values are very similar to the training set. This is evident by the low difference (<0.08, in the scale of relative likelihood of occurrence) between predictions based on actual predictor values and those obtained when predictor values were restricted to the range of the training set (the latter practice is called “clamping” in MaxEnt terminology). In fact, only the low altitude of Tawny Owls observed at the coasts of the Sea of Galilee was out of the training range. On the other hand, there are no recent Tawny Owls observations in some areas with high-predicted relative likelihood of occurrence, especially Judean Hills and Jerusalem (central Israel and The West Bank). Interestingly, historical evidence from mid-20th century implies that Tawny Owls occupied these areas in the past ([8,18] and references therein). The fact that Tawny Owls are more likely to occur in vegetation index values lower than 0.5 and higher than 2 (but less in values in between) suggests that Tawny Owls are likely to be found in maquis, but also in human settlements, but less likely in open fields and shrublands. The vegetation index’s unique contribution to the model (as suggested by the fact that its omission decreased the gain more than any other predictor) is corroborated by the fact that the climatic and topographical predictors are highly correlated in our study area, while vegetation density is also impacted by development and conservation policy.

The Tawny Owl is a generalist predator, with a relatively high percent of avian prey (12–29%), especially compared to other owls [14,30]. Despite being a Palearctic species at the southern edge of its distribution, Tawny Owl nesting success in Israel is adversely affected by severe winters. This may reflect prey scarcity, as Günther’s Vole (*Microtus guentheri*), the Tawny Owl’s main prey species in our study area, is limited to the Near East and north Africa [31], and hence might be more sensitive to cold weather than the Tawny Owl, which also occurs in high latitudes in Scandinavia.

Interestingly, the Tawny Owl’s natural history (breeding seasonality and success rate, diet pattern and habitat) in Israel is very similar to that reported from Europe (Table 6), despite the large difference in climate. This could be interpreted as the species’ limited ability to adapt its natural history to local conditions. On the other hand, the species’ sensitivity to cold spring temperatures in local terms (e.g., minimum temperatures at Safed were as low as 1 °C twice during March 1992; [32]), along with its ability to raise hatchlings when temperatures regularly top 30 °C, suggest some climatic adaptations in the local population. The mechanism that underlies these observations, such as annual weather’s impact on prey availability, warrants further research.

The Tawny Owls readily adopted nesting boxes, including those in rural settlements and agricultural settings. Combined with its vole-focused diet in rural habitats, these facts suggest that Tawny Owls may not only adapt to relatively close human association, but also thrive as a commensal, anthropophilic species [33] feeding on commensal prey. Solonen and af Ursin [34] found similar results in Finland, where Tawny Owl breeding success was high in both rural and urban habitats (~2.5 “nearly fully grown young” per nest; compare to ~1.7 hatchlings per nest herein). The notion that Tawny Owls may survive in Israel as commensals is further supported by our MaxEnt output that indicated high relative likelihood of Tawny Owl occurrence in low vegetation index values (i.e., settlements and their immediate vicinity). Tawny Owls were observed and even nested not only in villages, but also in densely populated cities such as Haifa and Safed.

The Tawny Owl’s southern edge of distribution shifted north from Hebron (Judean Hills, The West Bank; [35]) to Menashe Heights (just south of Mount Carmel, northern Israel). It is unlikely that this range shift was due to climate change, since a recent study in Israel found very little change in both precipitation and temperature means from the 1920’s until the 1990’s [36], while the last reported observation of Tawny Owl nesting from Judean Hills is from Jerusalem at January 1974 [37]. Moreover, attributing these extirpations to urban development is not obvious too, because Tawny Owls are regularly observed in European cities with no negative impacts on breeding success [34,38,39]. On the other hand, our results suggest that as the local climate warms and becomes more arid, breeding success will decrease. Taken together, our study suggests that while the Tawny Owl can survive rural development trends, climate change presents a real threat to its survival in its southern edge of distribution.

## Figures and Tables

**Table 1:** Summary of the splitting of training and test data of the MaxEnt (Maximum Entropy) models used to infer the relative likelihood of *Strix aluco* occurrence in the Upper Galilee. See text.

MaxEnt Model	Training Latitudinal Ranges	Training Observations	Test Latitudinal Ranges	Test Observations
Model A	32.981°–33.13° N	94	32.9°–32.981° N	32
Model B	32.981°–32.98° N 33.021°–33.13° N	95	32.981°–33.021° N	31
Model C	32.9°–33.021° N 33.047°–33.13° N	95	33.021°–33.047° N	31
Model D	32.9°–33.047° N	94	33.047°–33.13° N	32
Combined	32.9°–33.3° N	126	-	-

**Table 2:** Number of eggs and fledglings in Tawny Owl (*Strix aluco*) nests in the Upper Galilee, Israel.

Dataset	N	Eggs			Fledglings		
		Mean ± SD	Mode	Max	Mean ± SD	Mode	Max
No. of eggs laid is known and >0	105	3.152 ± 0.959	3	6	1.686 ± 1.470	0	5
At least 1 fledgling *	110	3.373 ± 0.671	3	5	2.391 ± 0.889	2	5
At least one egg or at least one fledgling *	148	-	-	-	1.777 ± 1.298	2	5

* These data include nests where the number of eggs laid was unknown. Max = maximum; SD = standard deviation.

**Table 3:** Summary of the nesting data by year.

Nesting Year	87′	88′	89′	90′	91′	92′	93′	94′	95′	96′	Total
Sites surveyed for eggs	5	30	47	40	42	26	22	19	20	17	268
Nests where the no. of eggs laid is known and >0 (% of surveyed)	5 (100%)	15 (50%)	16 (34%)	14 (35%)	14 (33%)	2 (8%)	10 (45%)	7 (37%)	9 (45%)	13 (76%)	105 (39%)
At least 1 hatchling *	0	9	6	10	9	2	8	5	6	12	67
Mean clutch size ± standard deviation *	3.0 ± 1.4	3.1 ± 1.0	3.1 ± 1.5	3.1 ± 1.2	3.1 ± 0.6	2.5 ± 0.7	3.3 ± 0.5	3.1 ± 0.4	3.1 ± 0.9	3.4 ± 0.7	3.2 ± 1.0
Mean brood size ± standard deviation *	0 ± 0.0	1.7 ± 1.7	1.0 ± 1.5	2.1 ± 1.5	1.6 ± 1.5	1.5 ± 0.7	1.7 ± 1.2	2.0 ± 1.5	1.9 ± 1.5	2.5 ± 1.1	1.7 ± 1.5
Total fledglings *	0	25	17	29	22	3	17	14	17	33	177

* In the nests where the number of eggs laid is known and greater than zero.

**Table 4:** Predictors of Tawny Owl (*Strix aluco*) nesting success in the Upper Galilee. Values are the coefficients in models of brood size, when positive coefficients indicate a positive correlation between the predictor and brood size and vice versa. The value of each ‘binary’ predictor was set to ‘true’ or ‘false’, depending on its dominance in the nest’s environment. Significant (α = 0.05) *p*-values are in **bold**. Significant negative coefficients are underlined while significant positive coefficients are in *italics*. The final model (right columns) was chosen by Akaike Information Criterion (AIC).

Predictor	Full Model		Final Model		
	Coefficient ± SE	*p*-Value	Coefficient ± SE	Standardized Coefficient	*p*-Value
(Intercept)	−2011 ± 4.763	**<0.001**	−2012 ± 4.593	–	**<0.001**
Pines (Binary)	*0.787 ± 0.375*	**0.036**	1.000 ± 0.280	**0.34**	**<0.001**
Oaks (Binary)	−0.303 ± 0.376	0.420	Omitted		
Rural (Binary)	−0.0004 ± 0.295	0.999	Omitted		
Number of hot days	−0.029 ± 0.046	0.618	Omitted		
Maximal seasonal temp. (C°)	−1.056 ± 0.122	**<0.001**	−1.094 ± 0.096	**−1.35**	**<0.001**
Minimal seasonal temp. (C°)	*2.859 ± 0.193*	**<0.001**	*2.901 ± 0.170*	**2.44**	**<0.001**
log10 (precipitation, mm)	*14.84 ± 2.177*	**<0.001**	*15.34 ± 1.837*	**1.26**	**<0.001**
AIC	427.58		422.17		

**Table 5:** Counts of Tawny Owl (*Strix aluco*) prey items in the Upper Galilee by taxon, summarized by dominant habitat type in the pellet collection site. “Maquis” is Mediterranean maquis, “pines” are *Pinus* spp. stands and “rural” means both small settlements (generally less than 1000 inhabitants) and agriculture. “NS” stands for non-significant.

Scientific Name	Common Name	Maquis	Maquis and Pines	Rural	Significantly More Common in
**Mammalia**	**Mammals (Total)**	**457**	**393**	**560**	
Soricidae	Shrews	37	27	18	Maquis
*Meriones tristrami*	Tristram’s Jird	36	36	13	Maquis and pines
*Microtus guentheri*	Günther’s Vole	219	180	401	Rural
*Cricetulus migratorius*	Gray Hamster	5	4	1	NS
*Apodemus* spp.	Field Mice	109	69	24	Maquis, maquis and pines
*Mus musculus*/*macedonicus*	House and Macedonian mice	43	68	41	Maquis and pines
*Rattus rattus*	Black Rat	7	9	59	Rural
*Spalax ehrenbergi*	Middle East Blind Mole Rat	1	0	3	NS
**Aves**	**Birds (Total)**	**62**	**45**	**232**	
*Gallus gallus domesticus*	Domesticated chicken (Arabian)	0	0	1	NS
*Scolopax rusticola*	Eurasian Woodcock	3	0	0	NS
Columbidae	Pigeons and doves	6	9	69	Rural
*Tyto alba*	Barn Owl	0	0	1	NS
*Caprimulgus europaeus*	European Nightjar	0	1	0	NS
*Dendrocopos syriacus*	Syrian Woodpecker	0	2	0	NS
*Turdus merula*	Common Blackbird	13	2	6	Maquis
*Turdus philomelos*	Song Thrush	2	0	0	NS
*Erithacus rubecula*	European Robin	2	0	1	NS
*Muscicapa striata*	Spotted Flycatcher	0	0	1	NS
*Garrulus glandarius*	Eurasian Jay	1	0	11	Rural
*Corvus cornix*	Hooded Crow	0	5	3	NS
*Passer domesticus*	House Sparrow	21	16	117	Rural
*Carduelis carduelis*	European Goldfinch	2	0	1	NS
Aves (indet.)	Unidentified birds	12	10	21	NS
**Insecta**	**Insects**	**1**	**3**	**19**	**Rural**
**Total Prey Items**		**520**	**441**	**811**	**1772**

**Table 6:** Comparison of the Israeli Tawny Owl population (this study) to conspecific populations from Europe (data from studies reviewed by [4,25,27]). For the Israeli population, the age range presented is the mean ± standard deviation, using both the minimal and maximal ages for each ontogenetic stage (see Methods).

Natural History Trait	Israel		Europe
	Minimum Age	Maximum Age	
Age at fledging (days)	23–32	28–37	32–37
Age at flying (days) ^a^	42–76	48–83	46–51
Age at adolescence (days) ^b^	83–102	89–104	92–97
Mean clutch size	3.2 ± 1.0 (n = 105)		3.4 ± 1.1
Mean brood size	1.7 ± 1.5 (n = 105)		2.8 ± 1.2
% eggs hatched	86% (n = 43)		66% (n = 818)
% hatchlings fledged	73% (n = 37)		96% (n = 509)
% eggs survived to fledging	86% × 73% = 63%		66% × 96% = 63%
% birds in diet ^c^	33%		13% (rural); 63% (urban-suburban)
Male calling season	Year-round, mostly January-February and April-May		Year-round, mostly October-November
Start of egg laying season	Mid-late-March		Mid-March
Common habitat	Various woods and forests; to a lesser extent, rural settlements and fields; rarely, in cities.		Various woods and forests; also in rural settlements and even cities

^a^ Taken for the Israeli population as the age at “developed mobility”. ^b^ Defined as the age when young no longer depend on parents for food. ^c^–Percentage calculated out of total live weight of vertebrate prey only. For Europe, mean of 10 studies from rural areas.

## Data Availability

Data are available in Supplementary Materials S1–S3.

## Supplementary Materials

The following supporting information can be downloaded at: https://www.mdpi.com/article/10.3390/ani12050641/s1, Supplementary information S1: Nesting database, Supplementary information S2: Ontogenetic data, Supplementary information S3: Diet database.
